# Gender differential in low psychological health and low subjective well-being among older adults in India: With special focus on childless older adults

**DOI:** 10.1371/journal.pone.0247943

**Published:** 2021-03-08

**Authors:** Ratna Patel, Strong P. Marbaniang, Shobhit Srivastava, Pradeep Kumar, Shekhar Chauhan, David J. Simon

**Affiliations:** 1 Department of Public Health and Mortality Studies, International Institute for Population Sciences, Mumbai, India; 2 Department of Mathematical Demography & Statistics, International Institute for Population Sciences, Mumbai, India; 3 Department of Population Policies and Programmes, International Institute for Population Sciences, Mumbai, India; 4 Paris 1 Pantheon-Sorbonne University, Paris, France; Institute of Economic Growth, INDIA

## Abstract

**Background:**

Gender and health are two factors that shape the quality of life in old age. Previous available literature established an associaton between various demographic and socio-economic factors with the health and well-being of older adults in India; however, the influence of childless aged is neglected. Therefore, the study examined the gender differential in psychological health and subjective well-being among older adults, focusing on childless older adults.

**Methodology:**

This study utilized data from Building a Knowledge Base on Population Aging in India (BKPAI). Psychological health and subjective well-being were examined for 9541 older adults aged 60 years & above. Descriptive statistics and bivariate analysis were used to find the preliminary results. Further, multivariate analysis has been done to fulfill the objective of the study.

**Results:**

Around one-fifth (21.2%) of the men reported low psychological health, whereas around one-fourth (25.5%) of the women reported low psychological health. Further, around 24 per cent of men and 29 per cent of women reported low subjective well-being. Results found that low psychological well-being (OR = 1.87, C.I. = 1.16–3.01), as well as low subjective well-being (OR = 1.78, C.I. = 1.15–2.76), was higher in childless older women than in childless older men. Higher education, community involvement, good self-rated health, richest wealth quintile, and residing in urban areas significantly decrease the odds of low subjective well-being and low psychological well-being among older adults.

**Conclusion:**

There is a need to improve older adults’ psychological health and subjective well-being through expanded welfare provisions, especially for childless older adults. Moreover, there is an immediate requirement to cater to the needs of poor and uneducated older adults.

## Introduction

Population ageing is a human achievement, reflecting the reductions in fertility and the improvements in survival associated with economic and social development and advances in public health and medicine [1]. However, a growing ageing population in any country carries enormous social, economic, and public health implications, including higher expenditure on pension and healthcare, the need for social security reforms, shrinking of the workforce, and shortage of active persons who can support dependent older adults [2, 3]. India’s older adult population (aged 60 years and above) is expected to grow from 8 per cent (around 92 million) in 2010 to 19 per cent (323 million) by 2050 [4, 5], and the elderly dependency ratio will rise dramatically from 0.12 to 0.31 during the same period [6]. Older age is a very vulnerable phase of life. As the emotional and physical health declines at a later age, the increasing dependency on the caregivers results in older adults being exposed to the risk of being neglected, abused, and mistreated [7]. At present, the older population in many countries is experiencing many life problems, of which deteriorating health is the main issue [6]. Health and wellbeing are the most important factor at the later ages; it is the most crucial factor in predicting life satisfaction and wellbeing of the aged [6].

### Gender differences in psychological health and subjective wellbeing

Gender and health are two factors that shape the quality of life in old age [8]. Literature has highlighted the impact of gender on the health of older adults in India. Evidence has shown that a higher proportion of older women in India reported poor self-rated health and lower rates of good self-rated health as compared to men [2]. Women in India are more likely to be involved in unpaid domestic work due to social and cultural factors [9], making them more vulnerable to poor health outcomes [10]. Subjective well-being represents people’s evaluation of their lives based on cognitive and emotional reactions. The concept of subjective well-being (SWB) refers to the absence of mental illness and refers to a person’s optimum psychological functioning and experience [11]. Previous research has identified social relationships, social capital, socioeconomic status, and psychological resources as significant factors for SWB among older adults [12]. Apart from the above factors, gender is another important aspect determining older adults’ subjective well-being [13]. Even though in many societies across the world, women have a longer life expectancy than men, still women tend to report higher levels of distress, depression, and chronic illness [2]; this is because a large part of women additional years are spent with illness and disability [14]. Also, women tend to rank themselves low than men on emotional and cognitive measures of general well-being, such as self-rated health, life satisfaction, and will to live [15].

### Childless older adults and their psychological health and subjective wellbeing

Ageing without children is one of the well-documented research areas in developed countries, probably due to low fertility rates. However, this domain remains elusive from the grip of researchers in the Indian context [16, 17]. Studies related to ageing and childlessness among Indian older adults did not receive attention because it is very uncommon for Indians not to have children [18]. Previous studies have established a relationship between various demographic and socio-economic factors with the health and well-being of older adults in the Indian population [2, 19, 20]; however, the influence of childless aged is neglected. Society faces demographical aging due to fertility decline and increased longevity. As the older population rises, the need for social support and personal care also grows. Children are the most important social support source for older parents in emotional, financial, and other support [21]. The literature on care and hands-on help consistently mentioned the importance of adult children, especially daughters, as primary social support resources in old age [22]. However, given this importance, childlessness during old age affects life quality and required more attention [23]. According to some studies, childlessness during older age is associated with reduced well-being, loneliness, and an increased risk of geriatric depression [24]. Regarding gender, evidence suggests that childlessness during old age impacts men and women differently [24, 25]. Conflicting facts show that subjective well-being is higher among the childless elderly, or if they had children, satisfaction increases after children have left home [21].

To the best of our knowledge, limited studies have investigated gender differential on low psychological health and low subjective well-being among older adults in India, focusing on childless older adults to date. In view of the issues discussed earlier, this paper aims to examine the gender differential in psychological health and subjective well-being among older adults with a particular focus on childless older adults. This paper hypothesis that there would be no gender differences in psychological health and subjective well-being among older adults in India.

## Materials and methods

### Data

The study used data from Building a Knowledge Base on Population Aging in India (BKPAI), a national-level survey conducted in 2011 across India’s seven states. The survey gathered information on aging’s socio-economic and health aspects among those aged 60 years and above. Seven major regionally representative states were selected for the survey with the highest 60+ years population than the national average. This survey was carried out on a representative sample in India’s northern, western, eastern, and southern parts following a random sampling process. Details on the sampling procedure are available in national and state reports of BKPAI, 2011 [26]. For the current study, the effective sample size was 9541 older adults residing in seven states aged 60+ years.

#### Outcome variables

*Psychological Health*. Psychological health was measured with twelve questions based on the General Health Questionnaire Scale (GHQ-12). The questions were asked on four points Likert scale. The twelve questions included in the General Health Questionnaire (GHQ) are as follows.

1. Have you recently been able to concentrate on whatever you’re doing?
2. Have you recently lost much sleep due to some worry?
3. Have you recently felt constantly under strain?
4. Have you recently felt that you couldn’t overcome your difficulties?
5. Have you recently been feeling unhappy and depressed?
6. Have you recently been losing confidence in yourself?
7. Have you recently been thinking of yourself as a worthless person?
8. Have you recently felt that you are playing a useful role in life?
9. Have you recently felt capable of making decisions about things?
10. Have you recently been able to enjoy your normal day-to-day activities?
11. Have you recently been able to face up to your problems?
12. Have you recently been feeling reasonably happy, all things considered?

Psychological health was measured on a scale of 0 to 12 based on experiencing healthful symptoms. It was recoded as 0 “high” (representing six and above scores) and 1 “low” (representing score five and less) [27, 28]. The scale was in progressive order, but the responses were recoded and made binary, as mentioned in the above section. Further, the variables were scaled from 0–12 and recoded as per literature to make it binary for analytical purposes.

*Subjective well-being*. Subjective well-being was measured with the help of nine questions answered on a three-point Likert scale. The nine questions included in subjective well-being are as follows:

1. Do you feel your life is interesting?
2. Compared with the past, do you feel your present life is?
3. On the whole, how happy are you with the kind of things you have been doing in recent years?
4. Do you think you have achieved in your life the standard of living and the social status that you had expected?
5. How do you feel about the extent to which you have achieved success and are getting ahead?
6. Do you normally accomplish what you wanted to accomplish?
7. Do you feel you can manage situations even when they do not turn out to be as expected?
8. Do you feel confident that in the case of a crisis (anything that substantially upsets your situation in life), you will be able to handle it or face it boldly?
9. The way things are going now, do you feel confident in coping with your future?

Subjective well-being was measured on a scale of 0 to 9. It was categorized as 0 “high” experiencing better experience (representing six and above scores) and 1 “low” experiencing negative experience (representing score five and less) [29]. The scale was in progressive order, but the responses were recoded and made binary, as mentioned in the above section. Further, the variables were scaled from 0–9 and were recoded as per literature to make it binary for analytical purposes.

#### Predictor variables

The explanatory variables were categorized as per the literature cited in the introduction section. Having children(yes and no) was the main explanatory variable. Other predictors included age (60–69, 70–79 and 80+), gender (men and women), educational status (not educated, below five years, 6–10 years and 11+ years), marital status (not in a union and currently in a union), working status (last one year) (no, yes and retired), community involvement (no and yes), trust over someone (no and yes), living arrangement (alone, with spouse, with children and others), self-rated health (good and poor), wealth quintile (poorest, poorer, middle, richer and richest), religion (Hindu, Muslim, Sikh, and others), caste (Scheduled Caste/Scheduled Tribe (SC/ST) and non-SC/ST), residence (rural and urban) and states (Himachal Pradesh, Punjab, West Bengal, Orrisa, Maharashtra, Kerala, and Tamil Nadu).

### Statistical analysis

Descriptive statistics and bivariate analysis were used to find the preliminary results. Further, multivariate analysis (binary logistic) has been done to fulfil the objective of the study. The results were presented in an odds ratio (OR) with a 95% confidence interval (CI).

The model is usually put into a more compact form as follows:

$$\ln\left(\frac{P_{i}}{1-P_{i}}\right)=\beta_{0}+\beta_{1}x_{1}+\cdots +\beta_{M}x_{m-1},$$

Where *β*_0_,…..,*β_M_* are the regression coefficient indicating the relative effect of a particular explanatory variable on the outcome. These coefficients change as per the context in the analysis in the study. We examined the collinearity using the variance inflation factor (VIF). As it was found that VIF was not above 10 for any factor, so we proceeded with the analysis. Additionally, to find the gender differentials for psychological health and subjective well-being, the interaction effect was utilized. Moreover, model-1 was the unadjusted model observing interaction effect, model-2 was the full effect model, and model-3 was the adjusted model observing interaction effect.

## Results

**Fig 1:**
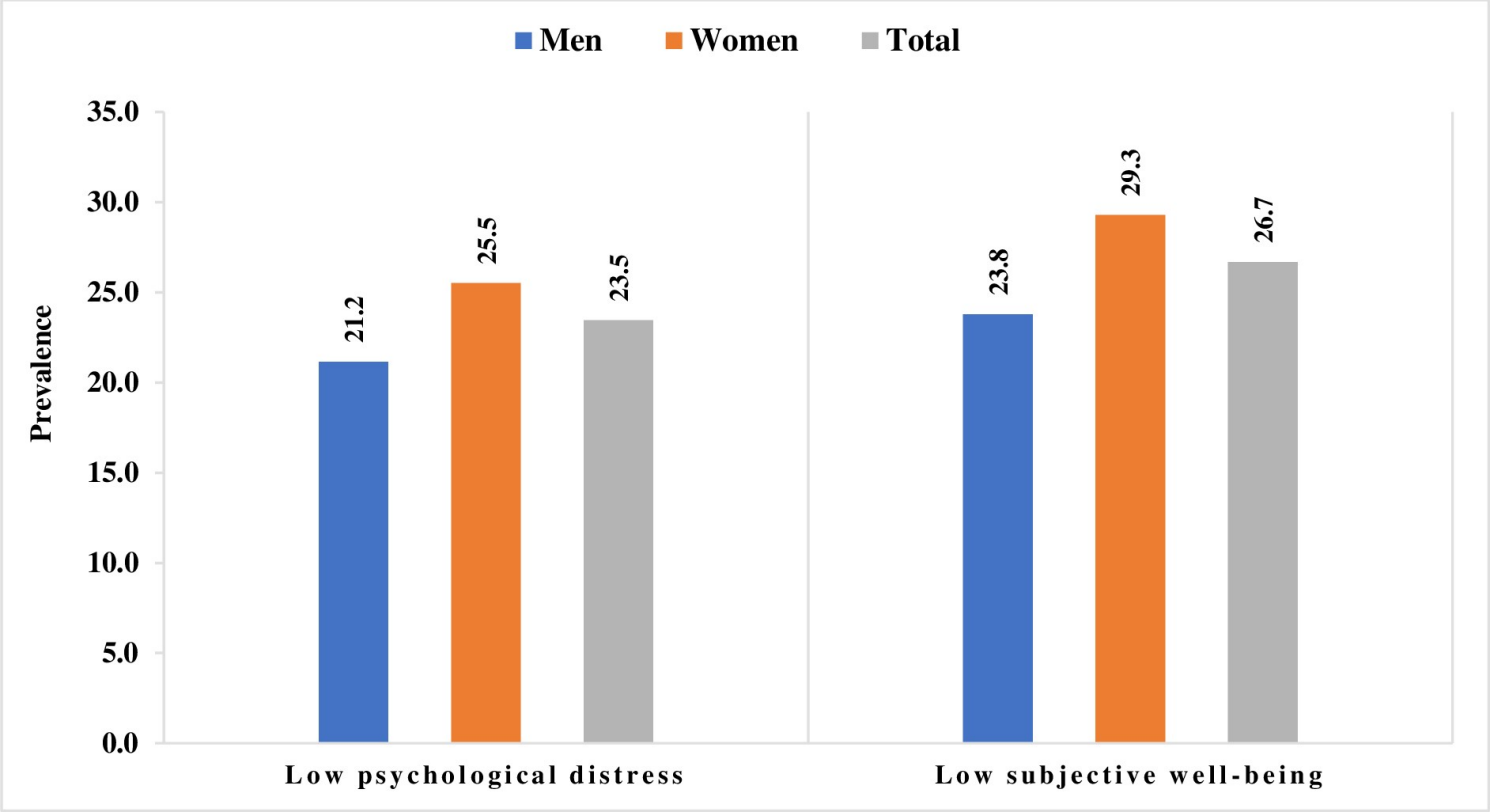
Prevalence of low psychological health and low subjective well-being among men and women.

Fig 1 shows the prevalence of low psychological health and low subjective well-being among men and women. A higher percentage of women reported low psychological distress as well as low subjective well-being than men. Around one-fifth (21.2%) of the men reported low psychological distress, whereas around one-fourth (25.5%) of the women reported low psychological distress. Around 24 per cent of men and 29 per cent of women reported low subjective well-being.

The socio-economic and demographic profile of the study population was presented in Table 1. Nearly three per cent of men and five per cent of women older adults did not have any child. A higher proportion of older adults belonged to the 60–69 years of age group, and most older adults were illiterate (men-34.2% and women-65.5%).

**Table 1:** Socio-economic and demographic profile of the study population, India.

Background characteristics	Men		Women	
Having children	Sample	Percentage	Sample	Percentage
Yes	4,185	96.6	4,557	94.8
No	149	3.4	250	5.2
**Age (years)**				
60–69	2,686	62.0	2,965	61.7
70–79	1,180	27.2	1,331	27.7
80+	468	10.8	511	10.6
**Educational status**				
Not educated	1,482	34.2	3,149	65.5
Below 5 years	998	23.0	890	18.5
6 to 10 Years	1,437	33.2	634	13.2
11+ years	417	9.6	135	2.8
**Marital status**				
Not in union	645	14.9	2,912	60.6
Currently in union	3,689	85.1	1,895	39.4
**Working status**				
No	1,944	44.9	4,210	87.6
Yes	1,675	38.7	524	10.9
Retired	715	16.5	73	1.5
**Community involvement**				
No	678	15.7	1,195	24.9
Yes	3,656	84.4	3,612	75.1
**Trust over someone**				
No	637	14.7	942	19.6
Yes	3,697	85.3	3,865	80.4
**Living arrangement**				
Alone	79	1.8	448	9.3
With spouse	920	21.2	549	11.4
With children	3,067	70.8	3,418	71.1
Others	269	6.2	392	8.2
**Self-rated health**				
Good	2,090	48.2	2,005	41.7
Poor	2,244	51.8	2,802	58.3
**Wealth quintile**				
Poorest	973	22.5	1,176	24.5
Poorer	934	21.6	1,082	22.5
Middle	881	20.3	1,016	21.1
Richer	855	19.7	841	17.5
Richest	689	15.9	691	14.4
**Religion**				
Hindu	3,481	80.3	3,781	78.7
Muslim	277	6.4	373	7.8
Sikh	405	9.3	444	9.2
Others	171	3.9	209	4.3
**Caste**				
Scheduled Caste	919	21.2	977	20.3
Scheduled Tribe	232	5.4	278	5.8
Other Backward Class	1,580	36.5	1,765	36.7
Others	1,603	37.0	1,787	37.2
**Place of residence**				
Rural	3,237	74.7	3,528	73.4
Urban	1,097	25.3	1,279	26.6
**State**				
Himachal Pradesh	717	16.5	745	15.5
Punjab	591	13.7	653	13.6
West Bengal	527	12.2	584	12.2
Orissa	737	17.0	708	14.7
Maharashtra	580	13.4	649	13.5
Kerala	564	13.0	757	15.8
Tamil Nadu	617	14.2	711	14.8
**Total**	4,334	100.0	4,807	100.0

Association of low psychological health and low subjective well-being by childless ageing and other socio-demographic characteristics were presented in Table 2. Results depict that women older adults with no children reported significantly higher low psychological health (34.1% vs. 20.7%) and low subjective well-being (40.4% vs. 28.6%) than men older adults. These two indicators were highly prevalent among the older adults who belonged to 80+ years of age, irrespective of gender. There was a negative association between older adults’ education and low psychological health, and low subjective well-being. Older adults currently in the union reported less low psychological health (men-20.2% and women-19.8%) and low subjective well-being (men-22.7% and women-22.9%) than those not in a union. The percentage of low psychological health and low subjective well-being was higher among older adults who had no community involvement and no trust over their counterparts.

**Table 2:** Association of low psychological health and low subjective well-being by childless ageing and other background characteristics, India.

Background characteristics	Low psychological health		p-value	Low subjective well-being		p-value
	Men (%)	Women (%)		Men (%)	Women (%)	
**Having children**						
Yes	21.2	25.1	0.001	23.6	28.7	0.001
No	20.7	34.1	0.001	28.6	40.4	0.010
**Age (years)**						
60–69	18.4	21.0	0.001	20.8	25.2	0.001
70–79	23.1	30.6	0.001	25.7	33.7	0.001
80+	31.8	38.6	0.225	35.9	41.9	0.036
**Educational status**						
Not educated	30.9	30.7	0.805	35.9	35.5	0.216
Below 5 years	23.4	21.1	0.040	26.6	20.9	0.002
6 to 10 Years	13.2	10.0	0.129	13.6	13.7	0.656
11+ years	8.3	8.0	0.785	9.3	12.3	0.155
**Marital status**						
Not in union	26.5	29.2	0.367	30.1	33.5	0.028
Currently in union	20.2	19.8	0.673	22.7	22.9	0.4061
**Working status**						
No	29.4	26.0	0.001	34.3	28.6	0.001
Yes	17.9	24.5	0.001	19.1	37.4	0.001
Retired	6.4	4.8	0.725	6.3	11.0	0.045
**Community involvement**						
No	32.9	35.7	0.249	39.4	40.6	0.364
Yes	19.0	22.2	0.001	20.9	25.6	0.001
**Trust over someone**						
No	35.4	38.8	0.146	42.9	42.6	0.376
Yes	18.7	22.3	0.001	20.5	26.1	0.001
**Living arrangement**						
Alone	39.5	31.7	0.538	33.3	39.8	0.314
With spouse	21.7	19.0	0.730	25.2	26.8	0.202
With children	20.3	25.3	0.001	22.8	27.7	0.001
Others	24.1	29.3	0.061	27.7	34.3	0.011
**Self-rated health**						
Good	11.3	12.5	0.026	12.8	16.8	0.001
Poor	30.4	34.9	0.003	34.0	38.3	0.001
**Wealth quintile**						
Poorest	35.9	38.0	0.159	43.8	49.8	0.026
Poorer	27.6	31.4	0.147	31.9	32.8	0.137
Middle	18.2	21.0	0.317	19.9	22.0	0.095
Richer	12.2	16.8	0.011	10.9	18.2	0.001
Richest	6.6	12.3	0.001	5.5	13.1	0.001
**Religion**						
Hindu	23.5	27.7	0.001	25.8	30.7	0.001
Muslim	17.2	27.1	0.005	25.3	32.8	0.010
Sikh	8.2	7.7	0.506	9.2	15.3	0.010
Others	9.8	21.3	0.044	13.9	26.9	0.001
**Caste**						
Scheduled Caste	25.1	30.9	0.005	31.6	36.1	0.010
Scheduled Tribe	31.9	33.1	0.355	32.1	37.5	0.0157
Other Backward Class	24.3	26.6	0.002	25.3	29.9	0.007
Others	14.2	20.4	0.001	16.6	23.7	0.001
**Place of residence**						
Rural	22.9	27.2	0.006	25.5	30.7	0.001
Urban	16.0	21.0	0.001	18.6	25.4	0.001
**State**						
Himachal Pradesh	13.0	20.9	0.002	13.0	16.6	0.003
Punjab	7.4	7.3	0.982	8.7	14.0	0.002
West Bengal	26.8	31.6	0.049	42.7	53.3	0.001
Orissa	35.1	39.8	0.011	33.9	36.5	0.212
Maharashtra	20.6	24.3	0.133	28.5	39.3	0.001
Kerala	8.7	17.3	0.001	11.4	16.9	0.004
Tamil Nadu	34.3	37.8	0.289	29.3	33.8	0.254
**Total**	21.2	25.5	0.001	23.8	29.3	*

Psychological health: General Health Scale (coded in binary form, i.e., low “scores five or less” and high “scores more than equal to six”)

Subjective well-being: Subjective Well-Being (coded in binary form, i.e., low “scores of five or less” and high “scores more than equal to six”)

Results from logistic regression for low psychological health and low subjective well-being were presented in Table 3.

**Table 3:** Estimates from logistic regression analysis for low psychological health and low subjective well-being by various background characteristics, India.

Background characteristics	Low psychological health			Low subjective well-being		
	Model-1	Model-2	Model-3	Model-1	Model-2	Model-3
	UOR (95% C.I)	AOR (95% C.I)	AOR (95% C.I)	UOR (95% C.I)	AOR (95% C.I)	AOR (95% C.I)
**Having children**						
Yes		Ref.			Ref.	
No		1.17(0.89,1.55)			1.38***(1.06,1.79)	
**Age (years)**						
60–69		Ref.	Ref.		Ref.	Ref.
70–79		1.27***(1.12,1.45)	1.27***(1.12,1.45)		1.30***(1.15,1.48)	1.30***(1.15,1.48)
80+		1.67***(1.38,2)	1.66***(1.38,2)		1.69***(1.41,2.02)	1.69***(1.41,2.02)
**Gender**						
Men		Ref.			Ref.	
Women		0.83*(0.72,0.95)			0.94(0.82,1.08)	
**Educational status**						
Not educated		Ref.	Ref.		Ref.	Ref.
Below 5 years		0.75***(0.65,0.87)	0.75***(0.65,0.87)		0.76***(0.65,0.87)	0.76***(0.65,0.87)
6 to 10 Years		0.49***(0.41,0.59)	0.49***(0.41,0.59)		0.55*(0.46,0.65)	0.55***(0.46,0.65)
11+ years		0.46***(0.33,0.65)	0.46***(0.33,0.65)		0.5***(0.36,0.69)	0.5***(0.36,0.69)
**Marital status**						
Not in union		Ref.	Ref.		Ref.	Ref.
Currently in union		0.98(0.85,1.13)	0.98(0.85,1.13)		0.97(0.84,1.11)	0.97(0.84,1.11)
**Working status**						
No		Ref.	Ref.		Ref.	Ref.
Yes		0.79***(0.68,0.92)	0.79***(0.68,0.92)		0.79***(0.68,0.92)	0.79***(0.68,0.92)
Retired		0.52***(0.38,0.7)	0.52***(0.38,0.7)		0.49***(0.37,0.66)	0.49***(0.37,0.66)
**Community involvement**						
No		Ref.	Ref.		Ref.	Ref.
Yes		0.71***(0.62,0.81)	0.71***(0.62,0.81)		0.66***(0.58,0.75)	0.66***(0.58,0.75)
**Trust over someone**						
No		Ref.	Ref.		Ref.	Ref.
Yes		0.75***(0.64,0.86)	0.75***(0.65,0.87)		0.64***(0.56,0.74)	0.64***(0.56,0.74)
**Living arrangement**						
Alone		Ref.	Ref.		Ref.	Ref.
With spouse		0.69***(0.52,0.92)	0.7***(0.53,0.94)		1.04(0.79,1.37)	1.04(0.79,1.38)
With children		0.96(0.75,1.22)	0.96(0.75,1.23)		1.05(0.83,1.34)	1.05(0.83,1.34)
Others		1.15(0.85,1.56)	1.16(0.86,1.57)		1.15(0.86,1.55)	1.15(0.86,1.55)
**Self-rated health**						
Good		Ref.	Ref.		Ref.	Ref.
Poor		3.91***(3.44,4.45)	3.91***(3.44,4.45)		3.14***(2.79,3.54)	3.14***(2.79,3.54)
**Wealth quintile**						
Poorest		Ref.	Ref.			
Poorer		1.08(0.92,1.28)	1.08(0.92,1.28)		0.79***(0.67,0.92)	0.79***(0.67,0.92)
Middle		0.94(0.77,1.13)	0.94(0.77,1.13)		0.58***(0.48,0.7)	0.58***(0.48,0.7)
Richer		0.79***(0.64,0.98)	0.79***(0.64,0.98)		0.51***(0.41,0.62)	0.51***(0.41,0.62)
Richest		0.64***(0.49,0.82)	0.64***(0.5,0.82)		0.34***(0.27,0.44)	0.34***(0.27,0.44)
**Religion**						
Hindu		Ref.	Ref.		Ref.	Ref.
Muslim		1.18(0.94,1.49)	1.18(0.94,1.49)		1.16(0.93,1.45)	1.16(0.93,1.45)
Sikh		0.98(0.66,1.44)	0.98(0.66,1.44)		1.11(0.79,1.56)	1.11(0.79,1.56)
Others		0.98(0.71,1.36)	0.98(0.71,1.36)		1.09(0.81,1.48)	1.09(0.81,1.48)
**Caste**						
Scheduled Caste		Ref.	Ref.		Ref.	Ref.
Scheduled Tribe		0.89(0.69,1.15)	0.89(0.69,1.15)		0.85(0.66,1.09)	0.85(0.66,1.09)
Other Backward Class		0.78***(0.66,0.92)	0.78***(0.66,0.92)		0.95(0.81,1.11)	0.95(0.81,1.11)
Others		0.85***(0.72,0.99)	0.85***(0.72,1.00)		0.81***(0.69,0.94)	0.81***(0.69,0.94)
**Place of residence**						
Rural		Ref.	Ref.		Ref.	Ref.
Urban		0.95(0.84,1.08)	0.95(0.84,1.08)		1.15***(1.02,1.3)	1.15***(1.02,1.3)
**State**						
Himachal Pradesh		Ref.	Ref.		Ref.	Ref.
Punjab		0.34***(0.24,0.48)	0.34***(0.24,0.48)		0.61***(0.45,0.84)	0.61***(0.45,0.84)
West Bengal		1.49***(1.19,1.87)	1.5***(1.19,1.87)		3.89***(3.11,4.87)	3.89***(3.11,4.87)
Orissa		2.38***(1.9,2.99)	2.38*(1.9,2.99)		1.96***(1.56,2.47)	1.96***(1.55,2.47)
Maharashtra		1.53***(1.22,1.92)	1.53***(1.22,1.92)		3.25***(2.6,4.05)	3.25***(2.60,4.05)
Kerala		0.72***(0.55,0.94)	0.72***(0.55,0.94)		0.93(0.72,1.21)	0.93(0.72,1.21)
Tamil Nadu		3.66***(2.87,4.66)	3.66***(2.87,4.66)		2.12***(1.66,2.7)	2.12***(1.66,2.7)
**Having child# Gender**						
No # men	Ref.		Ref.	Ref.		Ref.
Yes # men	0.91(0.61,1.36)		0.95(0.60,1.51)	0.73(0.51,1.05)		0.75(0.49,1.15)
Yes # women	1.17(0.78,1.73)		0.78(0.49,1.25)	1.01(0.70,1.45)		0.71(0.46,1.08)
No # women	1.87***(1.16,3.01)		0.97(0.55,1.68)	1.78***(1.15,2.76)		0.99(0.60,1.65)

***p<0.05; Ref: Reference; UOR: unadjusted odds ratio, AOR: adjusted odds ratio; CI: Confidence interval;#: Interaction

Model-1: Unadjusted model (Interaction)

Model-2: Adjusted model

Model-3: Adjusted model (Interaction)

Psychological health: General Health Scale (coded in binary form, i.e., low “scores five or less” and high “scores more than equal to six”)

Subjective well-being: Subjective Well-Being (coded in binary form, i.e., low “scores of five or less” and high “scores more than equal to six”)

### Low psychological health

*Model 1* represents the unadjusted interaction between gender and childless older adults for low psychological health. Moreover, *model 3* showed the adjusted results for the same. Older women who were not having a child (OR = 1.87, CI = 1.16–3.01, *Model 1*) were more likely to report low psychological health than older men who did not have any child; however, these results were not significant in the adjusted model (*model 3*). Age, gender, education, working status, community involvement, trust over someone, self-rated health, and wealth quintile were the significant predictors for low psychological health (*model 2*). Women older adults were 17 per cent (OR = 0.83, CI = 0.72–0.95, *model 2*) less likely to have low psychological health than men older adults. Higher education was linked to low levels of low psychological health. Older adults living with a spouse were 31 per cent (OR = 0.69, CI = 0.52–0.92, *model 2*) less likely to report low psychological health than older adults living alone.

### Low subjective well-being

On the other hand, model 1 showed unadjusted interaction between childless older adults and gender for low subjective well-being. Women older adults who did not have any child (OR = 1.78, CI = 1.15–2.76) were more likely to report low subjective well-being than men who did not have any child. This was also not significant when the study controlled other factors of the model (model 3). Age, education, working status, community involvement, trust over someone, self-rated health, wealth quintile, and place of residence were the significant predictors for low subjective well-being among older adults (model 2). Higher education is linked to low levels of subjective well-being among older adults. Older adults with poor self-rated health were 3.14 times more likely to have low subjective well-being levels than their counterparts.

## Discussion

This study examined gender differential in psychological health and subjective well-being among older adults with a particular focus on childless older adults. Previous studies highlighted psychological distress and subjective well-being among Indian older adults. However, limited research is attributed to the gender differential in psychological health and subjective well-being in relation to childless ageing [3, 30]. In the beginning, this paper hypothesized that there would be no gender differences in psychological health and subjective well-being among older adults in India. However, based on the study findings, we failed to find any support for our hypothesis, and therefore we have to reject our hypothesis. Results concluded that there is gender differential in psychological health and subjective well-being among older adults in India. This study highlights the higher prevalence of low psychological health and low subjective well-being among older women adults. Results from this study suggest considerable variations in low psychological health and low subjective well-being among older adults by selected socioeconomic characteristics such as age, gender, education status, working status, community involvement, trust, living arrangement, wealth, and caste. These socioeconomic variations in low psychological health and low subjective well-being have been documented in previous studies from India [31, 32]. The study did not find significant differences from interaction results between gender and having a child in an adjusted model. Previous studies in various settings have highlighted poor subjective well-being and psychological health among childless older adults disfavouring older women [33, 34]. However, a few studies did not find any significant association with gender [35].

Gender differences persist with various background characteristics, also disfavouring women older adults. The women’s disadvantages were observed in working status, living arrangement, self-rated health, wealth quintile, religion, caste, and place of residence. Previous studies also noted that women older adults tend to report a higher level of poor health statuses than men older adults [36]. Studies unanimously reported that women tend to live longer than men but expected to report being in worse health than men worldwide [36–38]. The fact that larger shares of women in India than men never attended school may partially explain how gender differences were more significant for women than their counterparts. Education has previously been an important factor in the study of gender disparity in health functions among older adults [39].

Education is one of the strongest predictors of low psychological health and low subjective well-being among older adults. The study noticed a negative relationship between education and these two variables. Higher education among older adults declines the odds of low psychological health and low subjective well-being. Available study noted an association between education and health as measured with self-rated health [40]. Higher education is strongly correlated with the overall quality of life as educated persons are more likely to be engaged in paid jobs, which further improve their psychological health and subjective well-being [41]. Furthermore, education has been hailed as a link that provides a better living standard that improves subjective well-being among older adults [42].

Wealth is one of the strongest predictors of low psychological health and low subjective well-being among older adults. Older adults in the richest wealth quintile were less likely to have low psychological health and low subjective well-being than the poorest older adults. Previous studies also highlighted the importance of wealth in achieving good psychological health and better subjective well-being among older adults [43]. These consistent associations between wealth and study variables are especially relevant given inconsistencies in previous research examining these relationships in smaller population groups [6, 44, 45]. The present study failed to document any significant association between religion and low psychological health along with low subjective well-being; however, Caste has emerged as a significant factor associated with low psychological health. These findings are consistent with previous studies in the Indian context [17]. In the Indian set-up, caste has been considered a proxy for socioeconomic status and poverty for a long [46]. Scheduled Castes and Scheduled Tribes (SCs and STs) have limited access to basic facilities and have lived under adverse conditions for centuries [47]. Also, the SC/ST population has a greater mortality risk across the life course than the higher caste group [48]. Similarly, access to education, proper nutrition, and basic healthcare among SCs and STs has been substantially lower than their counterparts [49]. Under the above-cited circumstances, there is a strong likelihood of low psychological health among SCs and STs compared to other caste groups [50].

Community involvement and trust have been positively associated with low psychological health and low subjective well-being among older adults. Various studies have highlighted the importance of trust and community involvement in reducing the odds of low psychological health low subjective well-being among older adults [51, 52]. Trust and community involvement provide a sense of security and comfort that further improves psychological health and subjective well-being among older adults [53]. Results found state-wise differential in low psychological health and low subjective well-being among older adults. As compared to older adults in Himachal Pradesh, older adults in Tamil Nadu were more than three times likely to report low psychological health and low subjective well-being. The odds were also lower in Punjab and Kerala. The results are consistent with a study in the Indian context [42].

The study has several limitations. The data was collected from seven states only. However, the population from these seven states was representative of the national sample [40, 42]. Furthermore, there are chances of misreporting of information, as the information on psychological health and subjective well-being was self-reported. Despite these limitations, the study has various strengths too. The present study adds to previous empirical evidence that women tend to have worse psychological health and subjective well-being, regardless of their socio-economic characteristics. Furthermore, the study adds a new dimension by examining childless ageing and its association with low psychological health and low subjective well-being among older adults in India.

## Conclusion

There is an implicit hypothesis based on previous studies that women tend to have poor psychological health and subjective well-being; this study confirmed that hypothesis. Furthermore, the study confirmed that childless ageing affects women more than men, as it was highlighted that childless older women were more prone to have low psychological health and low subjective well-being than childless older men. Moreover, gender differences were observed for various background characteristics too. The findings of this study have some potential policy implications. Firstly, it is important to carry out further studies in different relevant areas to identify various factors that may be related to psychological health and subjective well-being that may further lead to headway effective programs in improving older adults’ overall health conditions. Secondly, there is a need to improve older adults’ psychological health and subjective well-being through expanded welfare provisions, especially for childless older adults. Lastly, there is an immediate need to look out for vulnerable older adults like older adults who were poor and belonged to deprived caste groups and had no education.
