# Correction: Gender differential in low psychological health and low subjective well-being among older adults in India: With special focus on childless older adults

**DOI:** 10.1371/journal.pone.0271858

**Published:** 2022-07-18

**Authors:** Ratna Patel, Strong P. Marbaniang, Shobhit Srivastava, Pradeep Kumar, Shekhar Chauhan, David Jean Simon

The sixth author’s name is spelled incorrectly. The correct name is: David Jean Simon.
